# Adolescent engagement in a stepped care, transdiagnostic mental health intervention delivered in Indian schools

**DOI:** 10.1007/s44202-024-00154-1

**Published:** 2024-04-17

**Authors:** Resham Gellatly, Maya Boustani, Pooja Nair, Rujuta Mahajan, Abhijeet Jambhale, Rooplata Sahu, Bindiya Chodankar, Madhuri Krishna, Kanika Malik, Sonal Mathur, Kimberly Becker, Daniel Michelson, Vikram Patel, Bruce Chorpita

**Affiliations:** 1 Boston University Chobanian & Avedisian School of Medicine, Boston, MA USA; 2 Loma Linda University, Loma Linda, CA USA; 3 PRIDE Project, Sangath, New Delhi India; 4 Jindal School of Psychology and Counselling, O.P. Jindal Global University, Sonipat, Haryana India; 5 Department of Psychology, University of South Carolina, Columbia, SC USA; 6 Department of Child and Adolescent Psychiatry, Institute of Psychiatry, Psychology and Neuroscience, King’s College London, London, UK; 7 Department of Global Health and Social Medicine, Harvard Medical School, Boston, MA USA; 8 Department of Psychology, University of California, Los Angeles, CA USA

## Abstract

Given the relationship between poor engagement and worse treatment outcomes, improving engagement has been the focus of attention in recent years. Engagement is a particular challenge among minoritized and otherwise challenged youth, such as those from socioeconomically disadvantaged groups, including youth in low- and middle-income countries (LMICs), where they face lower levels of access to resources, including mental health treatment. The present study describes engagement challenges that arose in an uncontrolled pre-post evaluation of a school-based, modular, multi-problem, stepped-care intervention delivered in urban Indian communities. Specifically, the study aimed to (1) characterize barriers and facilitators of youth treatment engagement; and (2) evaluate treatment acceptability and fit of treatment from the youth perspective. Youth participants completed semi-structured interviews, which were transcribed and coded using thematic analysis. Participants described numerous facilitators to engagement (e.g., positive therapeutic relationship) and reported high overall satisfaction with the intervention, while also identifying barriers to engagement (e.g., concerns about confidentiality) and offering suggestions to increase fit and acceptability (e.g., more visually appealing treatment materials). Findings highlight ways in which engagement can be enhanced and implementation supports improved to maximize treatment effectiveness among minoritized and disadvantaged youth in LMICs.

**Supplementary Information:**

The online version contains supplementary material available at 10.1007/s44202-024-00154-1.

## Introduction

Improving engagement in children’s mental health services has long been a public health priority. Engagement has been defined as “an individual’s multidimensional (e.g., social, cognitive, affective, and behavioral) commitment to treatment, whereby dimensions exert reciprocal influence upon one another” [1]. Youth engagement in treatment is poor overall. Up to 50% of youth in need of services do not enter treatment [2], and among those who do initiate treatment, the majority terminate prematurely [3, 4]. Racial and ethnic disparities in rates of engagement are pronounced, with non-Hispanic White youth twice as likely to engage in treatment compared with youth with racial and ethnic minority identities [5]. Given the association between poor engagement and worse treatment outcomes [6–9], the need to improve youth engagement in treatment is an urgent priority. Accordingly, the past four decades have been marked by significant advances in the testing of implementation strategies to facilitate engagement in mental health services [1, 10–12].

Minoritized youth, a group that can be defined broadly and includes those with lower socioeconomic status, face substantial barriers to treatment engagement, both logistical (e.g., economic instability resulting in limited access to transportation; [13]) and perceptual (e.g., stigma, lack of trust in healthcare systems; [14]). Some of these barriers are hypothesized to stem from the historical development of evidence-based treatments (EBTs) in randomized controlled trials (RCTs) with primarily White participants, which raises questions about the cultural appropriateness of EBTs for various minoritized populations [15]. Further, the majority of these RCTs took place in the United States (U.S.), making the effectiveness of these EBTs uncertain when implemented in low- and middle-income countries (LMICs).

Compared with high-income countries, which have proportionately more researchers and resources available to develop and test mental health interventions, LMICs have been the setting for relatively few RCTs of psychological treatments [16, 17]. Additionally, the majority of investigations in LMICs have been limited to adult populations, with only a handful of studies examining interventions for children and adolescents (e.g., [18, 19]) and even fewer looking specifically at engagement processes. The dearth of research has resulted in disparities in access to culturally responsive treatments developed for youth in LMICs, an inequity even more pronounced within the subgroup of socioeconomically disadvantaged young people in LMICs, who face additional barriers to engaging in care as do minoritized youth in the U.S., rendering them vulnerable to living with untreated mental health problems for extended periods of time.

When engagement in youth interventions in LMICs has been described, its definition and measurement have largely been restricted to attendance and program completion [18, 20], though more recent studies have acknowledged that there may be indicators of poor engagement prior to missed sessions [21]. Attendance is an important indicator of treatment engagement and one that has also been shown to be associated with treatment outcomes [3, 22]. However, attendance alone is not sufficient to ensure positive treatment outcomes [3]. That is, although youth may be present in a session, active participation can be variable and can be impacted by numerous potential challenges to engaging meaningfully with the session content [23]. Thus, there is a need to understand better what characterizes treatment engagement in LMICs, especially in domains other than attendance.

Based on reviews [24, 25] of conceptual models of engagement proposed in the literature (e.g., [7, 26, 27], Becker and colleagues [1] posit that engagement is multidimensional, dynamic, and transactional such that the various dimensions exert a reciprocal influence upon one another and that these influences can change over time. They offer a measurement model, found to have strong structural validity [10], to characterize the literature on engagement interventions in children’s mental health. The REACH framework specifies five domains of engagement: Relationship (e.g., therapeutic alliance; [28]), Expectancy (e.g., beliefs that treatment will be helpful and that one can participate successfully in treatment; [29]), Attendance (e.g., presence at treatment sessions; [3]), Clarity (e.g., understanding about the treatment approach or the roles of each person involved in treatment; [30]), and Homework, which reflects multiple participation behaviors (e.g., homework completion, in-session participation; [3]). The conceptualization of engagement as multidimensional, dynamic, and transactional rather than unidimensional, static, and tied to the client alone differs from historical definitions of engagement and allows for a more nuanced and realistic understanding of the way engagement plays out in practice.

In additional to offering this conceptualization of engagement, Becker and colleagues conducted a review of 50 RCTs targeting youth engagement that revealed that certain practices were associated with improved engagement in each of the five REACH domains [1]. These findings suggest that engagement can be enhanced using specific interventions, at least within youth community mental health settings in the U.S. However, the scarcity of knowledge about youth engagement in treatment in LMICs, coupled with the need to promote child and adolescent mental health, underscores the urgency to understand what types of engagement challenges arise in low-resource settings to identify procedures to address challenges, enhance engagement, and improve clinical outcomes in this minoritized group.

The current study focused on understanding youth engagement in a stepped-care, multi-problem intervention for anxiety, depression, and conduct problems developed for adolescents in schools in India. The study characterized engagement barriers and facilitators that arose in a clinical case series (CCS) of the higher-intensity component of a stepped-care treatment (Step 2) within the PRemIum for aDolEscents (PRIDE) project. The PRIDE study took place in India from 2016 to 2022 with the goal of developing and testing a school-based psychosocial care model to address common adolescent mental health problems in India, including a stepped-care intervention composed of two sequential treatments of incremental intensity (Steps 1 and 2). Stepped care reserves more intensive treatments for individuals who do not benefit from initial, lower-intensity treatments, increasing accessibility and efficiency of evidence-based mental health care in low-resource settings [31]. Core practices included in the intervention were selected through systematic identification and matching of problems to practices based on the literature and the local context [32] and subsequently organized into a stepped care framework [33, 34]. The first step of the stepped-care architecture was Step 0, which consisted of school-wide sensitization activities to deliver psychoeducation on mental health and provide a referral pathway. Step 1 was a brief (4–5 session), low-intensity problem solving intervention guided by lay providers and supplemented by a printed comic with psychoeducational stories and self-completed practice exercises [35] that could also be delivered as a standalone intervention [36, 37]. Step 2 was a modular intervention that could be matched in real time to the main symptom presentations of non-responders to Step 1. The current study aimed to characterize barriers and facilitators of youth engagement in the Step 2 intervention organized according to the REACH framework.

## Methods

Qualitative methods were used to characterize engagement challenges from the youth perspective to inform revisions to the treatment protocol prior to an expanded evaluation of the intervention [38]. Data were collected between 2018 and 2019. All study procedures were approved by the Institutional Review Boards at the University of California, Los Angeles, Harvard Medical School, Sangath, and the Indian Council of Medical Research.

### Setting

The multi-site study was conducted in Delhi, India’s capital and the second largest urban area in the world, and Goa, a small state on India’s southwestern coast. In Goa, the study was conducted in six co-educational secondary schools supported by the Archdiocese Board of Education (ABE). Delhi schools included four senior secondary government-run schools: two co-educational, and one that ran in two shifts of all-boys and all-girls.

### Participants

Eligible participants for the study met the following criteria: (1) Non-response to Step 1 as defined by scoring at or above locally determined cutoffs of the Strengths and Difficulties Questionnaire (SDQ; [39]) Total Difficulties scale (> / = 19 for boys and > / = 20 for girls) and/or a score of 2 or greater on the SDQ Impact scale; (2) In grades 8–10 in Goa and grades 9–12 in Delhi. In total, we invited all participants who met these criteria (N = 32; 16 each from New Delhi and Goa) to participate in Step 2 with the aim of reaching the standard sample size for saturation for a relatively heterogenous study population given the differences between participants at the two sites [40]. We completed interviews and data collection with 19 participants (5 from New Delhi; 14 from Goa). The interviewed participants in Goa were on average 14.5 years (*SD* = 0.73, Range = 13–15), while the average age of Delhi students was 15.6 years (*SD* = 0.39, Range = 15–16). Study research staff obtained written informed youth assent and caregiver informed consent for youth under the age of 18 years. Youth over 18 years old provided written informed consent. Youth and caregivers had the option to decline research assent/consent while still participating in the treatment. Table 1 presents the youth sample characteristics.

**Table 1:** Youth characteristics

Youth characteristics^a^	Overall sample	Goa sample	Delhi sample
Gender *N* (%)			
Female	12 (63.16)	10 (71.42)	2 (40)
Male	7 (36.84)	4 (28.57)	3 (60)
Age, M(SD)	14.8 (0.81)	14.5 (0.73)	15.6(0.39)
Grade, *N* (%)			
Grade 8	3 (15.79)	3 (21.43)	0 (0)
Grade 9	13 (68.42)	9 (64.29)	4 (80)
Grade 10	3 (15.79)	2 (14.29)	1 (20)
Grade 11	0 (0)	0 (0)	0 (0)
Grade 12	0.(0)	0 (0)	0 (0)
Interview language			
English	8.(42.11)	8 (57.14)	0 (0)
Hindi	8.(42.11)	3 (21.43)	5 (100)
Konkani	3 (15.79)	3 (21.43)	0 (0)

^a^Youth interview paticipants

### Intervention

The Step 2 intervention was developed to be the most intensive treatment component of a school-based, stepped-care treatment for adolescents with common mental health problems (anxiety, depression, and conduct/anger) [33, 41]. The version of the working protocol for this study was a modular design that started with two compulsory modules (*Psychoeducation and Engagement*, *Relaxation*) for all youth; three behavioral modules (*Behavioral Activation*, *Exposure*, *Assertiveness and Communication*), a minimum of one that was selected by providers based on a combination of the youth’s reported top problems (YTP; [42]), scores on progress monitoring tools, and youth preference; two optional modules (*Cognitive Coping*, *Problem Solving*) that were added if youth response to the behavioral module(s) was suboptimal; and finally, a consolidation module, *Maintenance and Termination*, delivered to all youth. Modules were designed to span two to three sessions, although providers had the flexibility to spend more or less time in a module depending on a student’s demonstrated understanding of and proficiency with the concepts and skills. Protocol materials were developed collaboratively with the intervention design teams based at Sangath, India; the University of California, Los Angeles; and institutions in the U.K. Materials included a provider-facing manual, a flipbook with provider- and student-facing components, illustrated handouts for students to complete in session and at home, and appendices with supplementary guides. In the current study, Step 2 was delivered by qualified psychologists. In later iterations of the treatment, Step 2 was delivered by non-specialist providers [38]).

### Measures

*Youth interview guide.* Semi-structured interviews were conducted with students when they finished the Step 2 treatment. The youth interview guide was adapted from an existing interview guide that was used to explore participants’ experiences in a randomized controlled trial of Step 1 (see [35]). The interview included open-ended questions organized into five sections: (1) Initial engagement (e.g., “What were your initial thoughts when you first heard about the counseling service?”); (2) Relationship with providers (e.g., “Please tell me about your experience with your counselor”); (3) Experience with Step 2 content and materials (e.g., “How did the activities you learned relate to your problem?”); (4) Impacts of the intervention (e.g., “In what ways, if any, has counseling helped to bring about changes to your problems?”); and (5) Areas for improvement/experience ending counseling (e.g., “What other suggestions do you have about how to improve the counseling service for young people like you?”). Interviewers used non-leading probes such as “Tell me more about that,” and “Can you share an example?” to elicit additional information about youth experiences. Two study staff translated the student interview guide for use with students whose preferred language was Hindi.

### Interview procedure

Two Goa-based research staff were trained by the first author (RG) to conduct the semi-structured interviews with students in Goa. The training consisted of didactics on interviewing techniques (e.g., how to use probes to gather additional information), modeling, and a role-play of the entire interview. Three research staff conducted interviews in Delhi. Participants were offered the option of completing the interviews in their preferred language. Student interviews were conducted in English (*n* = 8); Hindi (*n* = 8); and Konkani (*n* = 3), with interviewers fluent in these languages. All interviews were audio-recorded. Hindi interviews were transcribed and translated into English by a professional Delhi-based agency; English interviews were transcribed by the same agency. The three Konkani interviews were translated into English and transcribed by a multi-lingual researcher with prior experience of translating and transcribing qualitative research interviews. Three members of the study team (RG, PN, and RM) cross-checked the transcriptions against the original audio recordings to verify accuracy.

### Coding and analysis

Interview transcripts were coded using an inductive-deductive approach to qualitative thematic analysis [43]. All interviews were coded using Dedoose, a qualitative data analytic software program. The first author (RG), who served as master coder, generated an initial set of a priori codes and definitions based on the interview guide, reflecting a deductive approach to coding. This taxonomy was applied to two student transcripts, which were coded by the master coder. Two undergraduate research assistants were trained in coding theory and procedures and assigned the same two transcripts to code independently. Coded transcripts were compared with the master coder’s coding. Discrepancies were discussed in a meeting, and codes and definitions were refined after reaching consensus. The remaining student transcripts were divided among the two coders to be coded independently. Coders were encouraged to identify potential new codes, which were discussed amongst the coding team in weekly meetings and incorporated into the codebook if consensus was reached on the value of their inclusion. This inductive coding process allowed for emergent codes not captured in the initial coding framework to be included. All coded transcripts were reviewed by the master coder, and discrepancies were resolved through discussion (see Appendix A for the coding framework).

Once all transcripts were coded, two senior members of the study team (RG and MB) synthesized codes into categories, themes, and sub-themes using qualitative thematic analysis [43], independently reviewed excerpts and identified key themes, and met to compare, discuss, and decide on final themes for inclusion. Study researchers engaged in reflexivity [44] as a core practice during the data collection, coding, and analysis stages of the project to consider how their individual identities and experiences impacted the research process throughout.

## Results

### Qualitative themes

Appendix B presents qualitative results from student interviews, including categories, themes, and sub-themes with additional exemplar quotes. Student responses fell into four broad categories of (a) Facilitators to treatment engagement; (b) Barriers to treatment engagement; (c) Impacts of the intervention; and (d) Recommendations for improvement. Descriptions of these categories, key themes and sub-themes are discussed below and organized according to the REACH (Relationship, Expectancy, Attendance, Clarity, Homework) engagement framework [1].

### Facilitators to treatment engagement

Students described facilitators to treatment engagement that map onto the REACH framework, including strong therapeutic alliance with counselors, activities built into the intervention that increased positive expectancy and improved clarity, and homework activities that were acceptable and shared with others.

#### Relationship

*Positive relationship with counselor facilitated treatment engagement*. The therapeutic relationship emerged as one of the most prominent facilitators of treatment engagement. Students expressed positive feelings toward their counselors and shared that they could trust their counselors and felt understood. One student in Goa, a state in which Christians comprise the second largest religious grouping after Hindus, said, “When I saw present counselor, it seems that she is like a God to me, more than my father and mother. I thought I can speak to her about anything just like I can with God.” Some students noted that they were sad about not seeing their counselors once treatment ended and expressed a desire to stay in contact.

*Theme 1.1. Youth perceived counselors as friendly, which strengthened their receptiveness*. Counselors were frequently described as friendly, nice, and happy, and students shared that these characteristics helped to put them at ease and strengthen their belief in the counselor and the intervention process. The way counselors spoke and explained concepts emerged as one distinct facilitator. One student said, “Actually, how she is explaining is nice, and I can share everything to her, and because of that she is telling what to do, how to do, if there is any problem there you can talk with others … Because how she is talking, I am feeling very nice.” Another said about their Step 2 counselor, “She was talking very friendly.” Counselors’ warmth and friendliness was also noted to have a positive impact on engagement, as a student who shared that she liked her counselor because of, “Her smile, and like she is very happy person so it was like very comfortable and I was thinking I can talk with her comfortably, I can share my problem.”

*Theme 1.2. Youth appreciated counselors taking time to learn about their interests*. The process of rapport building emerged as a facilitator of treatment engagement. Students reported enjoying discussing their interests with their counselors, such as the student who stated, “She spoke lovingly. She wanted to know more about me. I tried to tell as much as possible.” These conversations were seen as a way for counselors to obtain information about students’ problems in a non-threatening manner. One student stated, “She was very friendly. We didn’t directly start discussing with the problem, like she told me where she was from, and I was asking about her, then she was asking about my likes, dislikes, and then suddenly she was asking me like, ‘You like this subject?’ or ‘What you want to do?’ That way she came to know about my problem, and I told her, which she didn’t directly discuss. I found that she was very friendly and kind.”

#### Expectancy

Sensitization activities (Step 0) and positive peer experiences with counseling increased positive treatment expectancy and facilitated engagement.

*Theme 2.1. Expectation that counseling will help solve problems*. Sensitization activities centered around the idea that counselling can help students learn skills to solve their problems—consistent with the content of the brief problem-solving intervention that made up the first step of the stepped care intervention. This message stuck with interviewed students, who shared a belief that counseling would solve their problems. Some students explained that initially, they believed counselors would solve their problems for them, such as the student who reflected on their expectation that, “They can solve our problem.” Another student recalled thinking, “I think that I will try, I will share my problems with the counselor teacher, and I thought that it will work like Miss said.” It is possible that these positive expectations about counseling paved the way for students to self-refer and engage in treatment.

*Theme 2.2. Youth expectancy was impacted by others’ support*. Initial treatment engagement was facilitated by friends and family who recognized and shared the benefit of counseling, often through their own treatment experiences or positive expectations. Positive peer experiences in counseling were an especially powerful motivator of youth engaging in treatment. Students explained that their friends enjoyed counseling, which made them eager to experience it for themselves. As one student said, “All my friends love counseling, so I went for counseling.” Students also reported that seeing counseling work for their friends was a factor in seeking treatment, like the student who stated, “All my friends were going there because all they were having problem. Because their problems were solved, that is why also I went to the counseling.” Finally, students’ friends who were already in counseling explicitly encouraged the respondents to try it out. One student recalled, “My friends were going first, and they told me to go, and they will sort my problems.”

Parental approval of counseling also helped youth feel comfortable engaging. Students who were open with their parents about seeking treatment described receiving support, such as one student who shared, “I told my family members that I have gone there for counseling. My father told me that you should have told the counselors that you get angry easily.” Another student explained that it was important for his father to know that he was engaging in treatment because if he had not told him, “Then there will be tension that I am doing it without informing.”

#### Clarity

The sensitization videos and accompanying discussions in Step 0 were reported by students to increase their understanding of counseling and promote self-referral to treatment. Students also described their recognition of the applicability of skills taught to their problem as promoting engagement. Relatedly, progress monitoring was reported to be a motivating factor for students, demonstrating its role in helping to clarify students’ treatment goals and progress toward goals.

*Theme 3.1. Sensitization activities increased awareness and positive perceptions of counseling.* Whole-school sensitization activities were carried out at the beginning of the CCS to provide psychoeducation about services to school staff and students and generate appropriate referrals [35]. School principals and teachers were briefed on the purpose of counseling, including the types of students for whom counseling might be helpful. Teachers were asked to speak with students they felt could benefit from counseling before referring them to the study team. Classroom-based sensitization activities for students included counselor-delivered psychoeducation about services via an animated video, followed by a guided group discussion during which students were encouraged to ask questions and raise concerns. Students described the short video shown in the classroom sensitization session as relevant to their problems. One student said, “I liked the video a lot. I also understood [counseling] and I thought that I will like that.” Students also reported that the video portrayed counseling in a positive light and facilitated engagement in services. As one student explained, “I had seen the video, and I knew that counseling is not something bad.”

*Theme 3.2. Skills taught were relevant and enjoyable.* Students largely reported that the skills they learned were relevant to their problems and fun to learn and practice. One student who self-referred for difficulties with anger reflected on learning assertiveness and communication skills through roleplays with her counselor: “I used to fight a lot with my sister. I would talk with her in aggressive manner, and when I talked in an aggressive manner, then she, too, would talk in an aggressive manner. Miss [counselor] used to give example like my sister. She would talk and I would pretend to be shouting at her. She would be me and I would be my sister. Then she would talk and show and tell me that if I spoke to my sister like this then my problems would be reduced.” One student noted that Step 2 was tailored to her problems, in contrast to her experience of the Step 1 brief problem-solving intervention being more general. She stated, “In a half session only, I understood whatever [Step 2 counselor] tried to explain to me, but after a long session with [Step 1 counselor], it was like everything was something not of use … So, I wanted something which was based on my problem, which [Step 2 counselor] said.”

*Theme 3.3. Enjoyed tracking improvement *via* progress monitoring tools*. Students’ progress was tracked using three tools: a bar graph version of the Youth Top Problems measure, a mood rating using “smiley” emojis, and the Session by Session (SxS) version of the Strengths and Difficulties Questionnaire. Students reported that it was motivating to see their problems decrease throughout treatment, as the student who said, “Every session she would give me this. She would always ask me, ‘How much better do you think you will be in one month’s time?’ and I would say, ‘I always hope for a great day,’ and that actually happened. These papers [measures] are not just papers, actually, because they made me understand myself, which is a very great thing because we should be self-confident, which I was not at first, but these helped me to know myself.” Students also shared that they liked the bar graph format of the YTP. One student said, “First when I saw this, I was like, ‘What to do?’ I just hate to write. I thought she is going to tell to write. If I have to write, then it is going to be more boring. She told me that you can just draw the [YTP] graph. I started to draw that. That helped me. First day my anger was in 10, but at the last day of counseling, when we were closing our counseling, my anger was in 2. I can see this, and I can remember about that.”

#### Homework

Youth reported collaboratively selecting skills to practice and described introducing content to friends and family outside of sessions, suggesting that the practices were transferable and that students did not perceive them as stigmatizing.

*Theme 4.1. Youth collaboratively selected activities to practice*. Three relaxation activities were taught in the mandatory relaxation module, one of which students selected to practice more in depth. Students varied in their preference of the three activities, with students reporting liking one or more relaxation practices depending on perceived relevance, ease of use, and fun. A student explained why she preferred Happy Place to deep breathing: “It was like my biggest problem is concentration. I can’t concentrate and remember things because negative thoughts are just going all around my brain. So, Happy Place was something after a good imagination, after opening my eyes I would go and do my work, whatever I have to do.” Conversely, another student felt that deep breathing was more accessible than Happy Place, explaining, “Deep breathing was most helpful because at certain times you are sad and all you can’t just think about Happy Place. It takes quite a lot of time to keep yourself at corner and be with yourself doing Happy Place. Deep breathing you can do anywhere, so it was like very adjustable and comfortable for everything.”

*Theme 4.2. Youth practiced skills and shared content with friends and family*. Students reported that they discussed with family and friends their experience in counseling. One student reported practicing skills at home with her siblings: “One thing I shared with my sisters was exercise of relaxation of muscles and deep breathing. One sister was asking where I learned that. I told her that one of the teachers gave me this exercise. So, we did nicely. Three sisters did nicely. We were sitting in triangle shape and did this exercise. If there is some new exercise, we like to share our things.” Some students taught skills to their friends, as the student who stated, “I taught this muscle relaxation and deep breathing to my friends also. They loved it. They are doing now also sometimes.” A student noted that sharing takeaways helped them synthesize information, explaining, “Talking to them made me realize what was I doing in the counseling. So psychologically it will make me remember things because as they asked me things so I would tell them, and they remember that I did this in this counseling.”

### Barriers to treatment engagement

Students endorsed barriers to engagement that fit the REACH framework, such as negative treatment expectations related to stigma, lack of clarity about the purpose and structure of counseling, and materials perceived as needing improvement.

#### Relationship

*Theme 5. 1. Concerns about working with a new counselor.* The transition from Step 1 to Step 2 presented a barrier to engagement, particularly as it related to students establishing a relationship with their new Step 2 provider. One student explained her initial reservations, stating, “I was used to [Step 1 provider]. I was thinking how will [Step 2 provider] behave with me, whether she will understand my problem properly or I will be able to tell her my problem, but in first session only it was cleared, and whether she get angry or not that was cleared by this second session.”

#### Expectancy

While some students expressed holding positive expectations about counseling shaped by their peers’ openness in sharing their personal experiences, other students described negative expectations related to concerns about stigma.

*Theme 6.1. Worries about stigma from friends/family made students hesitant to be open about seeking counseling.* Although less commonly reported than expected based on the literature on stigma as a prevalent barrier to treatment in LMICs [45], several students stated that they were wary of coming to counseling and being open about seeking treatment because of concerns about stigma. Per one student’s report, “Friends and all will tell each other ‘He is going to a counselor,’ and would make fun of me if I shared with them.” Another described general negative perceptions of counseling, explaining, “I don’t know but everyone thinks that going to counseling is a foolish thing. I don’t know why.”

#### Attendance

Treatment sessions were held during the school day, which reportedly presented a problem for students who had concerns about missing class and negatively impacted attendance.

*Theme 7.1. Sessions interfered with classes, which was especially problematic for older students*. Some students felt that coming to sessions was a challenge because they had to miss class. For students in more advanced grades who faced increasing academic demands and pressure, missing classes for treatment was not a viable option. As one student explained, “Now I will take off my name from counselor sessions because I am in class 10, and I want to concentrate on my studies. Yes, I am in 10th standard [grade] and studies are tough. I don’t want to take risk as students often get failure in this class.” Another shared that they did not get enough time in counseling because of competing academic demands: “It was not that much, 30 min per session … It was less because we have all this lecture … Also, because I had final exams, that’s why.”

Attendance was also impacted by the setting of intervention delivery. Discrepancies in rates of treatment completion between the Delhi and Goa sites was significant. Treatment was delivered as planned in schools in Goa, while in Delhi, there was a shift from school-based delivery to an off-campus clinic due to extenuating circumstances. This shift in setting likely contributed to only two out of 16 participants completing treatment in Delhi while in Goa, 14 out of 16 participants completed treatment. Youths’ reported reasons for dropping out were problem resolved (Goa: n = 1; Delhi: n = 1), competing school-related time demands (Goa: n = 1; Delhi: n = 3), and no longer interested in participating (Delhi: n = 7). In Delhi, two students did not respond to attempts to follow up, and one student declined to provide a reason. This finding demonstrates the utility of delivering services in schools and reducing barriers to access.

#### Clarity

For many students, counseling was a relatively unknown concept prior to the initial study rollout, and that lack of understanding presented a challenge to engagement. Students reflected on their initial expectations of counseling, including worries about their privacy being violated.

*Theme 8.1. Youth initially had a limited understanding of counseling that contributed to hesitations about engaging in treatment.* Students reported that their limited understanding of counseling and its potential benefits contributed to hesitations about seeking services. One student shared, “I was not sure of counseling but to tell our problems.” Another was not sure how counseling might provide additional support and skills to cope with their “tension,” a catchall phrase for anxiety and stress, recalling, “When they had come the first time [to provide information on counseling], I was in seventh or eighth standard [grade]. At that time, I thought I will not take. I had tension but I was not thinking about that. I was thinking that I can manage my own things. Afterwards when I came to ninth standard [grade], I had more stress about the co-curricular activities and so I had to do [counseling.]”.

*Theme 8.2. Concerns about confidentiality presented a barrier to initiating treatment*. Many students described confidentiality as a barrier to engaging in treatment. Concerns about parents, teachers, and friends finding out about their challenges came up frequently. One student said, “I was scared that they will inform at our home.” Another endorsed feeling fearful, stating, “I was feeling scared. I thought Miss will say something to someone else.” When students learned about confidentiality, they expressed relief: “I came to know from other kids that your issues will not be shared with anyone, and it will be a secret.” As students built relationships with their counselors, their worries about confidentiality were allayed, as the student who said, “I felt confident about her. I thought that she is trustworthy and will not disclose my things to anyone.”

#### Homework

While most students did not provide feedback on the materials, which included the student-facing flipbook and handouts, and largely described the materials as “helpful” and “easy,” several offered suggestions to improve the materials and facilitate their use outside of session.

*Theme 9.1. Overall satisfaction with materials but recommendation for improvement*. Students suggested incorporating more colors to make the materials “attractive” and to facilitate understanding, such as the student who offered, “Like red color means bad and yellow color means good. If a person is angry, it should be flagged as red and if a person is not angry it should be flagged as good and yellow. It would be easy for others to understand.” Another suggested having more blanks on the handouts to allow for practice: “These blank spaces, like there are only 8. It could be 15 instead so that children could do more after the session and all.”

### Impacts of the intervention

Students described positive treatment effects, including both symptom reduction and improvement in functioning across settings. They reported noticing changes in themselves and receiving feedback from others that suggested a positive benefit from counseling.

*Theme 10.1. Treatment resulted in functional improvement in youths’ lives*. Students shared that their lives changed as a result of participating in counseling. Across problem types, students reported that the skills they learned were relevant, transferable, and effective, even when they didn’t expect the skills to work, as the student who said, “It was like a very big difference in me, and I was thinking like it was small, small activities. I was thinking it won’t help me. First, I thought – I am telling you honestly – like I thought the counselors are good, but I thought these activities are like, stupid. I thought, ‘Let me try, let’s see what happens.’ I was trying, trying and it was like part of my life, and it started improving me and I noticed it.” Another student said simply, “I was getting angry earlier but now the anger is less. I am doing everything nicely in right time, right way.”

Students reported that their friends, family, and teachers also noticed positive changes. One recalled, “Teachers are also noticing that I am now quiet in class. Between when school started and now, they can see a difference. First, I was different; I kept talking [disruptively in class], not completing my work, and now I am like a good student.” Similarly, a student commented on her parents’ observation of her improved ability to control her anger: “My parents noticed, like when I got angry when I was watching TV and my father shut it down. Miss told me that I should start taking deep breaths, and my mother said ‘She didn’t get angry, something is there …’ I was talking nicely with my father. So, I said ‘This is because of counseling.’ So, she said, ‘Good, go for counseling if that happened because of the counseling.’” Importantly, students felt prepared to use skills on their own. One student summed it up: “I have learned new skills to decide on my own, like now I can decide what is right and what is wrong.”

### Recommendations

Students offered recommendations to improve the counseling experience, ranging from materials to treatment duration to different delivery formats.

*Theme 11.1. Desire for extended time with counselors*. Several respondents suggested increasing the session duration, frequency, or overall length of treatment to spend more time with their counselors. One student explained, “I want more time with my counselor, I want to sit alone with her and talk nicely. She also talks nicely.” Another stated, “It is like twice in a week is actually not okay. So at least make it thrice a week.”

*Theme 11.2. Activities can be improved in various ways, including having more options for practice and different formats*. Students suggested that the treatment include more “fun” and “accessible” activities, in addition to more options for relaxation practices. Changing the format of counseling was also a recommendation given by one student, who said, “One of the ideas is group counseling. It should be done with the friends and the people who have the same problems. It will make you think lesser. The same person will explain the whole lot of things to the same group. They will understand and it will be utilized. It will also save time.”

*Theme 11.3. Increase students’ privacy by changing the system for calling students and making materials more discreet*. Confidentiality was brought up as an essential element of treatment. Students were typically called to session by counselors receiving them from the classroom, which was noted as problematic by some. One student said that she did not like, “How counselor called students in the class loudly. There are other students, also, so that thing should be changed. That should be changed by giving note.” Making the handouts more discreet was offered as another recommendation to increase privacy: “If I take something in my hand and they are standing there and they read and say, ‘Hey, [student’s name] got the certificate as she is going for counseling.”

## Discussion

This qualitative study examined barriers and facilitators to treatment engagement from the perspective of young people who participated in a modular, multi-problem, school-based intervention for adolescents in Goa and Delhi, India. Youth participants also described the impact of treatment and offered recommendations. Although youth reported preliminary hesitations about initiating treatment, they described overall strong engagement in the intervention once treatment commenced. The therapeutic relationship emerged as a key facilitator to engagement as reported by youth, as did peer influence. Challenges were noted in relation to delivery in the school setting, such as competing academic demands and privacy. Youth discussed positive outcomes of participating in treatment, including functional improvements in their lives, while offering recommendations to increase acceptability and fit with the context. Notwithstanding these identified challenges, results suggest that students largely perceived the treatment to be engaging, acceptable, and appropriate for the context. These results align with quantitative evidence that the treatment is feasible, acceptable, and effective, with significant improvements on the SDQ Total Difficulties and Impact Scales, as well as the YTP [33]. Although there could be a selection bias given that students who chose to participate in the Step 2 intervention may have been more engaged with treatment from the start, the findings provide preliminary evidence to guide optimization and scale up of this multi-problem treatment across India, increasing adolescents’ access to evidence-based mental healthcare for a range of problems and narrowing the treatment gap in LMICs.

Many of the facilitators and barriers to treatment identified by youth are consistent with the literature on engagement. The current study offers a unique contribution through its application of the REACH framework [1] and its context within an LMIC, where limited research on youth engagement has been conducted. The REACH framework proposes five domains of engagement: Relationship, Expectancy, Attendance, Homework, and Clarity. Considering the Relationship domain, results from the current study underscore the power of a warm, caring, and collaborative relationship, in alignment with findings from previous research that highlight the impact that providers’ reactions, including verbal and non-verbal responses, can have on youth engagement [46] and prior outcomes from the PRIDE project [36]. Youth described providers’ characteristics, such as their smiles and manner of speaking, as important to them feeling comfortable, along with the perception that providers were genuinely curious about their interests and invested in their wellbeing. Students’ description of counselors as adults in whom they could speak freely as they do with God may reflect the counselors’ effort to take a non-judgmental stance. Student responses demonstrate that the time invested in building a solid relationship facilitated youth engagement and their positive perceptions of the treatment.

The role of expectancy, the second REACH domain, emerged as another significant component of youth engagement. Expectancy has been defined as the expectation that treatment will help and belief in one’s ability to participate successfully in treatment [29]. This attitudinal component of engagement has also been conceptualized as “buy-in” when combined with a client’s investment in, or commitment to, treatment [47]. When clients believe that treatment will be helpful and have realistic expectations about the treatment process, they are more likely to stay in treatment and have better outcomes [48]. Previous research has demonstrated that preparatory techniques such as providing information on treatment prior to initiating treatment facilitate realistic expectations that lead to better engagement [49]. Results from the current study support this finding. Students reported that school-wide sensitization activities in Step 0 increased their knowledge about counseling and led to the expectation that counseling would help them learn to solve their problems. Peers’ successful treatment experiences also facilitated youths’ positive expectations about treatment. The power of social influence is well-documented. Rogers’ [50] model of diffusion of innovations proposes that new ideas and practices are communicated and taken up among members of a social system, and that individuals are more likely to adopt an innovation when learning about it from a close and trusted source of knowledge, such as a close friend or relative. Youth responses in the present study substantiate this theory; youth reported that hearing about their friends’ experiences in therapy made them curious about the process and fostered a belief that treatment would be effective for them as well. Parental support also played a role in youths’ decision to seek treatment, with some describing how their parents’ encouragement helped them feel more comfortable participating in treatment. While preparatory techniques and peer influence increased youths’ positive expectations about treatment prior to initiation, youth shared that tracking their progress on measures at each session was motivating and provided concrete evidence that treatment was working. Given that attitudinal engagement can be conceptualized as a developmental process such that perceptions of treatment effectiveness during treatment might impact expectancy and therefore engagement [51], the potential benefit of youth-facing progress monitoring tools on engagement should be explored in future research.

Attendance, another REACH domain, has long been one of the primary metrics of assessing engagement, as it is a behavioral indicator that is relatively easy to measure. While attendance alone does not fully capture an individual’s engagement in treatment, it is a marker of participation and can signal poor engagement, most notably through premature termination from treatment, although inconsistent attendance is often a precursor and warning sign. In the present study, attendance as measured by early dropout from treatment differed greatly between the Delhi and Goa sites, likely due to the Delhi participant sample needing to see counselors at an off-campus clinic rather than in school due to an unexpected change. Students also commented on the tension between attending sessions and missing classes. This was particularly a challenge for more advanced students preparing for competitive entry exams for grades 11 and 12, known as higher secondary school in India. Although schools have been identified as an ideal setting in which to scale up delivery of mental health services in LMICs [52, 53], the challenges reported by students in the current study have been described as barriers in other studies [20] and should be addressed to allow youth to attend both treatment sessions and classes. Getting buy-in from school administrators and staff and involving them in the process of balancing these priorities is essential.

Most youth who participated in the intervention had limited knowledge about and experience with psychotherapy and described initial hesitations about participating rooted in lack of clarity about what to expect. Clarity as defined by the REACH framework encompasses an individual’s understanding of the treatment rationale, approach, structure, goals, and roles. Based on findings from previous qualitative research conducted in schools in Goa and Delhi during the preliminary formative phase of research of PRIDE [35, 54], the intervention development team aimed to increase clarity and address concerns about confidentiality through school-wide sensitizations that included psychoeducation about services in this phase of research. Despite these efforts, worries about confidentiality were raised by some youth in the current study and represented a potential barrier to engaging in treatment. This finding is consistent with the literature on the importance young people place on privacy and confidentiality in mental health treatment [55]. Youths’ desire for confidentiality related to their concerns about stigma, as they feared being teased or misunderstood if their participation in treatment were made known, though some students were more open about their connection to care. Continued efforts to increase knowledge about mental health and clarify what treatment entails might reduce the stigma of help-seeking and related worries about confidentiality. Clarity on the purpose of skills taught and their relevance to the problem was described as a facilitator to engagement. Youth largely perceived skills as helpful for their problems, demonstrating their awareness of treatment targets and how activities related.

Homework, the fifth domain of the REACH framework, represents an individual’s participation in treatment activities in and out of sessions. Completion of homework between sessions has been recognized as a means of facilitating skill acquisition and mastery and is associated with improved clinical outcomes across diagnoses [56, 57]. Practicing skills at home also gives individuals a chance to generalize therapy activities to real world settings and identify barriers to doing so [58] and has been hypothesized to increase self-efficacy [59]. Students in the current study reported practicing skills outside of session and teaching skills to family members and friends, suggesting a high level of engagement.

Recommendations included increasing the feasibility of at-home activities by taking into consideration fit for context and making them easier to practice outside the school setting, as well as revamping the design of practice materials to make them more colorful and attractive. These recommendations highlight the importance of collaborating with stakeholders to co-design treatment materials using a person-based approach [60]. Although providers were part of the design process from the early stages of protocol development through implementation [10], youth were less directly involved. The intervention development team used youth feedback from earlier phases of the project to inform material design, taking into consideration expressed preferences for high-quality graphics that were colorful, shorter text, and simpler language [36]. However, youth expressed that the materials used in the current study did not fully meet these benchmarks, perhaps in part because youth who participated in Step 2 had previously used the printed comic books used in the Step 1 intervention and may have been primed to expect similar content presented in that format [38]. This feedback points to a need for more frequent feedback from end users during the design process to ensure that the protocol and accompanying materials are as enjoyable, usable, engaging, and effective as intended, in alignment with previous findings that that designing for cultural context can yield robust rates of engagement [61]. Although students identified barriers to engagement and offered ways in which the intervention could be improved, the barriers that emerged were not related to intervention content, highlighting the overall acceptability of the treatment for youth participants [62]. Importantly, youth described the treatment as effective and leading to improvements in their lives.

## Limitations and future directions

The current study has a number of strengths, including its rich qualitative analysis of minoritized youth perspectives on the high-intensity component of a stepped care intervention in India. The findings support prior qualitative work within the PRIDE project, including several engagement challenges and facilitators found to be consistent between Step 1 and Step 2, and add new information to our understanding of engagement in the high-intensity component of a stepped care intervention. However, these findings need to be considered within the study’s limitations. The change in service delivery setting in Delhi is a significant limitation. The treatment was designed to be a school-based intervention, and barriers to engaging students arose when the setting was changed to an off-campus clinic. This challenge likely contributed to poor engagement of students in Delhi. Delhi students’ low rates of participation in the behavioral and optional modules represents another limitation, as youth perspectives on those later modules were reported primarily from Goa. Additionally, of the 19 youth whose interviews were included in analysis, only five were from Delhi despite the study team’s efforts to follow up with all Delhi students who had initiated treatment. It is possible that youth who dropped out prematurely were not motivated to engage with study staff or may have encountered other barriers, such as being on summer holiday by the time they were contacted about participating in interviews. Another limitation is that clinical problem type (e.g., anxiety) might be a potential factor influencing youth feedback, such that youth who were anxious may have overreported satisfaction with the treatment despite being interviewed by research staff who were not involved in treatment delivery.

To address these limitations, future research may focus on larger samples to test quantitative differences and examine how contextual factors (e.g., urban vs. semi-urban settings, language, culture) as well as participant characteristics (e.g., youth age) can impact engagement. Future investigations might also consider ways to follow up with hard-to-reach study participants to obtain their impressions of treatment by anticipating and addressing barriers that could interfere with their ability to be contacted. Additionally, the current study focused on youth perspectives of the intervention. Provider perspectives may shed light on barriers and facilitators from a different angle and offer pathways for increasing youth engagement. Providers delivering treatment in the current study were interviewed, and their responses will be analyzed and reported on in a more comprehensive examination of the intervention’s fit for the culture and context. Finally, future investigations might explore the effectiveness of using interventions identified in the evidence base that map onto the REACH framework to enhance engagement and address barriers described in the current study.

## Conclusion

The present study contributes to the relatively nascent literature on engaging youth in mental health care in LMICs. Although youth identified barriers to treatment engagement and highlighted areas for improving fit and acceptability, they also described numerous facilitators to engagement and reported high satisfaction with the intervention overall. Recommendations provided by youth are feasible, consistent with the literature, and offer avenues to improve engagement, bolster implementation supports, and enhance treatment effectiveness in minoritized populations in LMICs. Involving stakeholders in treatment design and being responsive to their needs and preferences paves the way for more culturally and contextually aligned interventions to be developed, implemented, and sustained within settings that have traditionally lacked the evidence base and resources to meet the needs of minoritized youth experiencing mental health challenges.

### Supplementary Information

Below is the link to the electronic supplementary material.

Supplementary file1 (DOCX 25 KB)

## Data Availability

The data presented in this study are available on request from the corresponding author. The data are not publicly available due to privacy.
