# Supplementary material for: Adolescent engagement in a stepped care, transdiagnostic mental health intervention delivered in Indian schools
Source: Discov Psychol. 2024 Apr 17;4(1):43. doi: 10.1007/s44202-024-00154-1 (PMC11057193; doi:10.1007/s44202-024-00154-1)
Supplement: Supplementary file 1 — Supplementary file1 (DOCX 25 KB) [file 44202_2024_154_MOESM1_ESM.docx]

## Supplementary Material.

## *Appendix A:* *Coding Framework*

| **Level 1 Codes** | **Level 2 Codes** | **Level 3 Codes** | |
| --- | --- | --- | --- |
| Initial Engagement | Awareness of PRIDE counseling services | Formal sensitization activities | |
|  |  | Peers | |
|  |  | Other | |
|  | Reason for referral (presenting problem) | High risk behavior/situation (suicidal ideation, self-harm, domestic violence) | |
|  |  | Other | |
|  | Mode of referral | Self | |
|  |  | Teacher | |
|  |  | Other | |
|  | Expectations of counseling | Get to discuss/share/solve problem | |
|  |  | Nervous | |
|  |  | Curious | |
|  |  | Not helpful | |
|  |  | Get to miss class | |
|  |  | Private/confidential | |
|  | Influence of others to initiate counseling | Peers | |
|  |  | Family | |
|  |  | Teachers | |
|  |  | Other | |
| Barriers to Initiating or Continuing Treatment | Confidentiality |  | |
|  | Scheduling/interference with class |  |  |
|  | Stigma |  |  |
|  | Literacy |  |  |
|  | Language |  |  |
|  | Other |  |  |
|  | Discussed with provider |  |  |
| Experience with Step 1 | Got to discuss problem |  | |
|  | Relationship with provider | Provider characteristics | |
|  |  | Understood problem | |
|  |  | Social desirability | |
|  | Time spent with provider |  | |
|  | Relevance to/impact on problem |  | |
|  | Use of skills outside session |  | |
|  | POD booklets |  | |
| Experience with Step 2 | Got to discuss problem |  | |
|  | Relationship with provider | Provider characteristics | |
|  |  | Understanding of problem | |
|  |  | Social desirability | |
|  | Relevance to/impact on problem |  | |
|  | Use of skills outside session |  | |
|  | Involvement of others in treatment |  | |
|  | Time spent with provider |  | |
| Step 1 vs. Step 2 | Comparison between providers |  | |
|  | Transition between steps |  | |
| Discussed Counseling with Others | Family |  | |
|  | Peers |  | |
|  | Teachers |  | |
|  | Others |  | |
| Experience with Step 2 Content | Progress monitoring | YTP | Positive |
|  |  |  | Negative |
|  |  |  | Neutral |
|  |  | Other | Positive |
|  |  |  | Negative |
|  |  |  | Neutral |
|  | Psychoeducation/Engagement | Positive | |
|  |  | Negative | |
|  |  | Neutral | |
|  | Relaxation | Deep breathing | Positive |
|  |  |  | Negative |
|  |  |  | Neutral |
|  |  | Happy Place | Positive |
|  |  |  | Negative |
|  |  |  | Neutral |
|  |  | Deep muscle relaxation | Positive |
|  |  |  | Negative |
|  |  |  | Neutral |
|  | Behavioral Activation | Positive | |
|  |  | Negative | |
|  |  | Neutral | |
|  | Assertiveness and Communication | Positive | |
|  |  | Negative | |
|  |  | Neutral | |
|  | Exposure | Positive | |
|  |  | Negative | |
|  |  | Neutral | |
|  | Cognitive | Positive | |
|  |  | Negative | |
|  |  | Neutral | |
|  | Problem Solving | Positive | |
|  |  | Negative | |
|  |  | Neutral | |
|  | Maintenance and Termination | Positive | |
|  |  | Negative | |
|  |  | Neutral | |
|  | Other |  | |
| Experience with Step 2 Materials | Flipbook | Positive | |
|  |  | Negative | |
|  |  | Neutral | |
|  | Handouts | Positive | |
|  |  | Negative | |
|  |  | Neutral | |
| Impacts of the Intervention | Positive |  | |
|  | Negative |  | |
|  | Neutral |  | |
|  | Plan to use skills in the future/belief in ability to solve own problems |  | |
|  | Belief that counseling is helpful |  | |
|  | Don’t remember |  | |
| Experience Ending Counseling |  |  | |
| Satisfaction with Treatment |  |  | |
| Suggestions |  |  | |

## *Appendix B:* *Qualitative Themes*

| **Facilitators to engaging in treatment** | **Theme** | **Select exemplar quotes** |
| --- | --- | --- |
| Relationship | Youth perceived counselors as friendly, which strengthened their receptiveness | “I liked her smile, and like, she is a very happy person so it was like very comfortable, and I was thinking I can talk with her comfortably, I can share my problem.” |
|  | Youth appreciated counselors taking time to learn about their interests | “She was very friendly. We didn’t directly start discussing with the problem, like she told me where she was from and I was asking about her, then she was asking about my likes, dislikes, and then suddenly she was asking me like, ‘You like this subject?’ or ‘What you want to do?’ That way she came to know about my problem, and I told her, which she didn’t directly discuss. So, I found that she was very friendly and kind.” |
| Expectancy | Expectation that counseling will help solve problems | “I will tell about my problem properly and then after I join it would be solved.” |
|  | Youth expectancy was impacted by others’ support | “All my friends love counseling, so I went for counseling.” |
| Clarity | Sensitization activities increased awareness and positive perceptions of counseling | “I liked the video a lot. I also understood [counseling] and I thought that I will like that.” |
|  | Skills taught were relevant and enjoyable | “It was something like, you know, looking for the negative, unhappy guessing. I would understand it better with the examples, whatever she gave. Like for example, for my problem, there was unhappy guessing or self-blaming. I always used to say, ‘I am bad, and I am not good. I am not good looking. I don’t have any friends.’ So, it was like Miss told to try out these exercises and then see. So, I tried, and I made some of my classmates as friends, and then later she asked me that how I am feeling now, and I said I am feeling good. A great thing about her was she would give me examples based on my own life.” |
|  | Enjoyed tracking improvement via progress monitoring tools | “Actually, you are filling that form, no? After that my problems were going less, less, less. And whenever I am filling this, I am feeling very happy because the problems are going less. |
| Homework | Youth collaboratively selected activities to practice | “When we think about happy place then my stress gets reduced, and yesterday Miss introduced me to deep breathing, so when I get angry, I do that.” |
|  | Youth practiced skills and shared content with friends and family | “One thing I shared with my sisters was exercise of relaxation of muscles and deep breathing. One sister was asking where I learned that. I told her that one of the teachers gave me this exercise. So, we did nicely. Three sisters did nicely. We were sitting in triangle shape and did this exercise. If there is some new exercise, we like to share our things.” |
| **Barriers to engaging in treatment** | **Theme** | **Select exemplar quotes** |
| Relationship | Concerns about working with a new counselor | “I was used to [Step 1 provider]. I was thinking how will [Step 2 provider] behave with me, whether she will understand my problem properly or I will be able to tell her my problem, but in first session only it was cleared, and whether she get angry or not that was cleared by this second session.” |
| Expectancy | Worries about stigma from friends/family made students hesitant to be open about seeking counseling | “Actually, I didn’t tell my parents that I am going to counseling. They will think that I am a fool. And my brother and sister are very young. So, they will not understand a word of this.” |
| Attendance | Sessions interfered with classes, which was especially problematic for older students | “Now I will take off my name from counselor sessions because I am in class 10th and I want to concentrate on your studies. Yes, I am in 10th standard and studies are tough. I don’t want to take risk as students often get compartment and failure in this class.” |
| Clarity | Youth initially had a limited understanding of counseling that contributed to hesitations about engaging in treatment | “I was not sure of counseling but to tell our problems.” |
|  | Concerns about confidentiality presented a barrier to initiating treatment | “I was afraid of it, actually, that my secret will be revealed to someone.” |
| Homework | Overall satisfaction with materials but recommendation for improvement | “The colors should be more attractive and in variations.” |
| **Impacts of the intervention** | **Theme** | **Select exemplar quotes** |
|  | Treatment resulted in functional improvement in youths' lives | “Life is changed. I was angry first. Because of my anger, my friends were not talking with me. Other friends were talking for five minutes or two minutes. They were not coming close to me and sharing anything. After doing that counseling, my anger came down. Everyone was coming and telling me about this. I did understand when they noticed that change. I knew how to control the anger. Then they say that I am not getting angry at all. I said I got angry but after knowing reason they are like teasing me and I told them I know how to control. Then they said, ‘Now we see how much you can control.’ These friends came close, parents came close, and teachers also came close to me.” |
| **Recommendations** | **Theme** | **Select exemplar quotes** |
|  | Desire for extended time with counselors | “I thought that they should keep it three days in a week. I love to go there and when she speaks, I just liked that style.” |
|  | Activities can be improved in various ways, including having more options for practice and different formats | “I think the counselor should tell like whether you are accessible to that activity like she told me playing and all, but I was not having that opportunity.” |
|  | Increase students’ privacy by changing the system for calling students and making materials more discreet | “How counselor called students in the class loudly. There are other students also, so that thing should be changed. That should be changed by giving note. You can call for the first counseling but in the next counseling, you can give note that these students should come. We can show that note to teacher and they can come for counseling.” |
